# *Trypanosoma brucei* TIF2 and TRF Suppress VSG Switching Using Overlapping and Independent Mechanisms

**DOI:** 10.1371/journal.pone.0156746

**Published:** 2016-06-03

**Authors:** Sanaa E. Jehi, Vishal Nanavaty, Bibo Li

**Affiliations:** 1 Center for Gene Regulation in Health and Disease, Department of Biological, Geological, and Environmental Sciences, Cleveland State University, Cleveland, Ohio, United States of America; 2 The Rockefeller University, New York, New York, United States of America; 3 Department of Immunology, Cleveland Clinic Lerner Research Institute, Cleveland, Ohio, United States of America; 4 Case Comprehensive Cancer Center, Case Western Reserve University, Cleveland, Ohio, United States of America; University of Texas Medical School at Houston, UNITED STATES

## Abstract

*Trypanosoma brucei* causes debilitating human African trypanosomiasis and evades the host’s immune response by regularly switching its major surface antigen, VSG, which is expressed exclusively from subtelomeric loci. We previously showed that two interacting telomere proteins, *Tb*TRF and *Tb*TIF2, are essential for cell proliferation and suppress VSG switching by inhibiting DNA recombination events involving the whole active VSG expression site. We now find that *Tb*TIF2 stabilizes *Tb*TRF protein levels by inhibiting their degradation by the 26S proteasome, indicating that decreased *Tb*TRF protein levels in *Tb*TIF2-depleted cells contribute to more frequent VSG switching and eventual cell growth arrest. Surprisingly, although *Tb*TIF2 depletion leads to more subtelomeric DNA double strand breaks (DSBs) that are both potent VSG switching inducers and detrimental to cell viability, *Tb*TRF depletion does not increase the amount of DSBs inside subtelomeric VSG expression sites. Furthermore, expressing an ectopic allele of F2H-*Tb*TRF in *Tb*TIF2 RNAi cells allowed cells to maintain normal *Tb*TRF protein levels for a longer frame of time. This resulted in a mildly better cell growth and partially suppressed the phenotype of increased VSG switching frequency but did not suppress the phenotype of more subtelomeric DSBs in *Tb*TIF2-depleted cells. Therefore, *Tb*TIF2 depletion has two parallel effects: decreased *Tb*TRF protein levels and increased subtelomeric DSBs, both resulting in an acute increased VSG switching frequency and eventual cell growth arrest.

## Introduction

*Trypanosome brucei* is a protozoan parasite that causes fatal African trypanosomiasis in humans and nagana in cattle. While proliferating in the extracellular spaces of its mammalian host, bloodstream form (BF) *T*. *brucei* is directly exposed to the host immune surveillance. However, *T*. *brucei* regularly switches its major surface antigen, VSG, thereby evading the host’s immune response [1].

There are more than 2,500 *VSG* genes and pseudogenes in the *T*. *brucei* genome [2,3]. Most are located within long *VSG* gene arrays at subtelomere regions of megabase chromosomes of *T*. *brucei* [3], and these *VSGs* are normally not expressed. In addition to eleven pairs of megabase chromosomes that contain all essential genes, *T*. *brucei* also has 4–5 intermediate chromosomes and ~100 copies of minichromosomes of only 50–150 kb [4,5]. Individual *VSG* genes are located at two thirds of minichromosome subtelomeres [2], which are not expressed but contribute to the large *VSG* gene pool for efficient VSG switching [5]. BF VSGs are expressed exclusively from subtelomeric VSG expression sites (ESs) [6,7], which are polycistronically transcribed by RNA polymerase I (RNAP I) [8] in a strictly monoallelic manner [9]. *VSG* is the last gene in any ES, located within 2 kb from the telomeric repeats and 40–60 kb downstream of the ES promoter [7]. There are 15 ESs in the *T*. *brucei* Lister 427 strain used in this study, but at any moment, only one ES is fully transcribed, resulting in a single type of VSG being expressed on the cell surface [9]. Most ESs are located on megabase chromosomes, but at least one ES is located on an intermediate chromosome [10].

VSG switching is an essential *T*. *brucei* pathogenesis mechanism enabling long-term *T*. *brucei* infections [1]. VSG switching occurs through two major pathways [11]. In an *in situ* switch, the originally active VSG ES becomes silent and a silent one becomes expressed, which does not involve gene rearrangements. Another major pathway for VSG switching is DNA recombination-based. In crossover (CO) or telomere exchange (TE), the active *VSG* gene and a silent subtelomeric *VSG* gene (in a silent ES or at a minichromosome subtelomere) exchange places, often together with their downstream telomere sequences [12]. No genetic information is lost in CO/TE. In gene conversion (GC), a silent *VSG* gene is duplicated into the active ES to replace the originally active *VSG* gene, which is subsequently lost [13]. When GC only encompasses the *VSG* vicinity, it is referred to as *VSG* GC. GC can also include most of the ES and even ES promoter regions, in which case it is referred to as ES GC. In many published studies, GC has been shown to be the most frequent event in VSG switching [14–19].

It has been shown that several proteins required for homologous recombination are important for VSG switching. At double strand break (DSB) sites, RAD51 binds the single stranded 3’ overhang following 5’ end resection and promotes strand invasion in DNA homologous recombination [20]. Deletion of *T*. *brucei* RAD51 and one of its paralogues, RAD51-3, significantly reduced the VSG switching frequency [21,22]. Deletion of BRCA2, a mediator facilitating the loading of RAD51 onto the single-stranded DNA [23], also decreased VSG switching frequencies [24]. On the other hand, deletion of Topoisomerase 3 alpha [15] and its interacting factor BMI1 [16] led to nearly 10 fold higher VSG switching frequencies, as the BLM-Topo3-BMI1 complex normally promotes resolution of double Holliday Junction and results in non-crossover events during homologous recombination [25,26].

How VSG switching is initiated and regulated is poorly understood. Recent studies have shown that inducing DSBs at 70 bp repeats located immediately upstream of the active *VSG* gene resulted in ~250 fold higher VSG switching frequencies [27]. However, although DSBs inside the active VSG ES are potent VSG switching inducers, they are also deleterious to cells and cause more than 85% of cell death (~85%, ~92%, and ~93% of cell death when DSBs are induced at ES promoter, between 70 bp repeats and the *VSG* gene, and downstream of the *VSG* gene, respectively) [28]. Therefore, maintaining subtelomere integrity is essential for *T*. *brucei* viability. Nevertheless, DSBs can be detected within the 70 bp repeats even in WT *T*. *brucei* cells, indicating that this is likely a key factor for VSG switching initiation [27]. Apparently, balancing subtelomere stability and plasticity is important for parasite survival, and factors that influence the amount of subtelomere DSBs will influence VSG switching frequency.

So far, *T*. *bruce*i TIF2 is the only protein that has been shown to influence the amount of subtelomeric DSBs [19]. *Tb*TIF2 is an intrinsic component of the *T*. *brucei* telomere protein complex [19]. It interacts tightly with the duplex telomere DNA binding factor, *Tb*TRF, which has been shown to play an essential role in maintaining the terminal telomere structure [29]. Telomere proteins are well-known for their roles in protecting the chromosome ends from illegitimate DNA processes including degradation, fusion, and recombination [30]. However, the functions of telomere proteins in maintaining subtelomere integrity are not clear. We found that depletion of *Tb*TIF2 resulted in a significant increase in the amount of DSBs at subtelomeres and subsequent elevated VSG switching frequency [19], demonstrating for the first time that telomere proteins also play important roles in maintaining subtelomere integrity. However, the underlying mechanism of *Tb*TIF2 function is not clear. Interestingly, we found that *Tb*TRF also suppresses VSG switching, and the telomeric DNA binding activity of *Tb*TRF is essential for this function [18]. Importantly, depletion of either *Tb*TIF2 or *Tb*TRF increases the amount of GC-mediated VSG switching events mostly involving the whole ES, suggesting that they may function in a same genetic pathway in influencing VSG switching [18,19]. In mammals, TIN2, the functional homologue of *Tb*TIF2 [19,31] interacts with both TRF1 and TRF2, which bind the duplex TTAGGG repeats [32–34] and are functional homologues of *Tb*TRF [18,29]. In addition, the interaction between TIN2 and TRF1 is essential for preventing TRF1 from being ADP-ribosylated by a TRF1-interacting factor, Tankyrase 1 [35,36], which releases TRF1 from the telomere DNA [37] and allows subsequent ubiquitination of TRF1 by SCF^Fbx4^ [38] followed by proteasome-mediated degradation [38,39]. Functions of TIN2, TRF1, and TRF2 are partly overlapping because TIN2 interacts with both TRF1 and TRF2 and is the central protein in the six-membered mammalian telomere complex called Shelterin [40].

Because *Tb*TIF2 interacts with *Tb*TRF, and because depletion of either protein leads to an acute increase in VSG switching frequencies with similar switching mechanisms and eventual cell death [18,19], we decided to examine whether these two proteins function in the same pathway, which will help reveal the underlying mechanism of *Tb*TIF2’s function in maintaining subtelomere stability. In this work, we found that depletion of *Tb*TIF2 decreased *Tb*TRF protein levels but did not affect its mRNA levels. We further found that *Tb*TRF was degraded by the proteasome upon depletion of *Tb*TIF2. However, depletion of *Tb*TRF did not increase the amount of DSBs inside the subtelomeric VSG ESs. Furthermore, expression of an ectopic *Tb*TRF WT allele delayed *Tb*TRF degradation, mildly improved cell growth, and partially suppressed the phenotype of elevated VSG switching frequency in *Tb*TIF2 RNAi cells. However, this did not suppress the phenotype of increased amount of subtelomeric DSBs in *Tb*TIF2 RNAi cells. Therefore, our observations indicate that increased amounts of subtelomeric DSBs and decreased *Tb*TRF protein levels are two independent and parallel consequences of *Tb*TIF2 depletion, both contributing to increased VSG switching frequencies and eventual cell growth arrest.

## Materials and Methods

### Chromatin IP (ChIP)

ChIP was performed exactly the same way as described in [18]. Briefly, Cells were fixed with 1% Formaldehyde for 30 min at room temperature and cell lysate was sonicated in a Bioruptor (Diagenode Corp.) at medium level for 6 cycles (30 sec on/30sec off per cycle) at 4°C. IP was carried out using protein G Dynabeads (Life Technologies) coupled with a *Tb*TRF antibody [29] followed by wash, elution, reverse crosslinking, and DNA isolation. ChIP products were hybridized with a TTAGGG repeat or a 50 bp repeat probe in slot blot Southern analysis.

### VSG Switching Assay

Switching assays were performed according to [18,41]. Detailed switching assay protocol is included in the supplemental materials.

### Ligation mediated PCR (LMPCR)

LMPCR were performed the same way as described previously [19,27]. Briefly, in each ligation reaction, 2 μg of genomic DNA was either treated or not treated with 2 μl of T4 DNA Polymerase (3000 U/ml, New England BioLabs) in the presence of 200 μM dNTP and then ligated with 10 μl annealed adaptor. Three 1:3 serial dilutions of the ligated products were prepared and used in subsequent PCR using Hotstart Platinum^®^ Taq DNA Polymerase (Life Technologies) and a touchdown PCR program.

### MG-132 treatment

*T*. *brucei* cells were incubated in medium with 25 μM of MG-132 for 6 hours before proteins were extracted for western analysis. For RNAi induced samples, MG-132 was added to the medium 6 hours prior to the time point of harvesting cells.

## Results

### *Tb*TIF2 protects *Tb*TRF from being degraded by the 26S proteasome

We observed a similar growth arrest phenotype upon RNAi induction in *Tb*TIF2 and *Tb*TRF RNAi cells [19,29]. In addition, a transient *Tb*TIF2 or *Tb*TRF RNAi induction resulted in a significant increase in VSG switching frequency with most VSG switchers arising from subtelomeric gene rearrangements that resulted in the loss of the whole active ES [18,19]. Therefore, we hypothesized that *Tb*TIF2 and *Tb*TRF function in the same genetic pathway in maintaining subtelomere stability.

**Fig 1:**
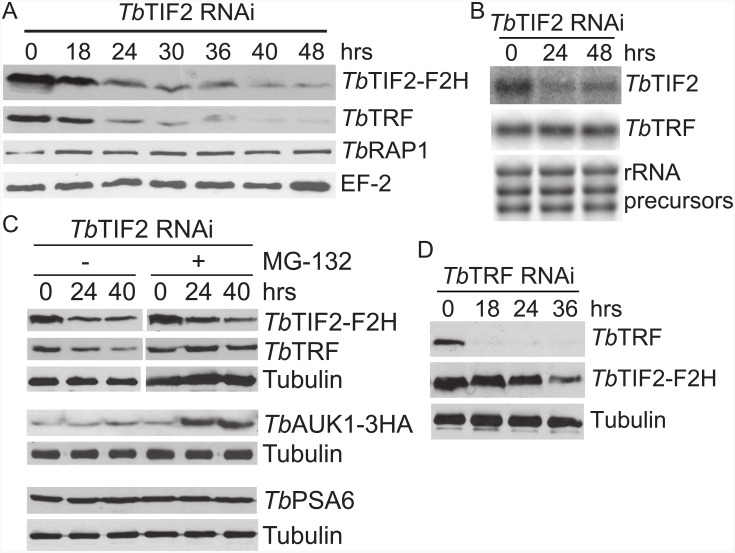
*Tb*TIF2 is essential for maintaining normal *Tb*TRF protein levels. (A) Western blotting was performed with whole cell extracts prepared at various time points after induction of *Tb*TIF2 RNAi (shown on top). (B) Northern blotting shows that *Tb*TRF mRNA levels were not affected in *Tb*TIF2-depleted cells. rRNA precursors were shown as a loading control. (C) Western blotting was performed using whole cell extracts prepared from *Tb*TIF2 RNAi cells treated with and without MG-132 (Sigma), an inhibitor of the 26S proteasome. *Tb*AUK1 is normally degraded by the 26S proteasome while *Tb*PSA6 is not. (D) Western analysis showed that *Tb*TIF2 protein levels are not sensitive to *Tb*TRF depletion. In western analyses, *Tb*TIF2-F2H and *Tb*AUK1-3HA were detected using an HA monoclonal antibody (F-7, SantaCruz Biotechnology). *Tb*TRF [29], *Tb*RAP1 [42], *Tb*PSA6 [43], and tubulin [44] were detected by their respective antibodies. EF-2 was detected using a goat polyclonal antibody against human EF-2 (Santa Cruz Biotechnology). All primary antibodies were diluted 1,000 fold. In this and other figures, proteins from 15 million cells were loaded each lane for western blotting, except when detecting tubulin, proteins from only 0.5 million cells were loaded each lane.

We first tested whether *Tb*TRF protein levels were affected by *Tb*TIF2. Indeed, induction of *Tb*TIF2 RNAi led to depletion of not only the endogenous FLAG-HA-HA (F2H) tagged *Tb*TIF2 (Fig 1A) but also the endogenous *Tb*TRF protein (Fig 1A). In contrast, protein levels of *Tb*RAP1, another intrinsic component of the *T*. *brucei* telomere complex [42], were not affected (Fig 1A). Northern blotting analysis showed that only the *Tb*TIF2 mRNA was knocked-down by *Tb*TIF2 RNAi, while *Tb*TRF mRNA levels were not affected (Fig 1B), indicating that *Tb*TIF2 is required for maintaining *Tb*TRF protein levels.

To investigate whether *Tb*TRF is degraded by the proteasome upon *Tb*TIF2-depletion, we treated *Tb*TIF2 RNAi cells with and without MG-132, which is a specific proteasome inhibitor and has previously been successfully used in *T*. *brucei* [45]. We found that in cells not treated with MG-132, *Tb*TIF2 was depleted upon induction of RNAi (the relative levels of *Tb*TIF2-F2H are 100%, 81%, and 79% at 0, 24, and 40 hrs after induction, respectively), which led to a quick *Tb*TRF depletion (Fig 1C). In contrast, in cells treated with MG-132, although *Tb*TIF2 was again depleted by RNAi at a very similar rate (the relative levels of *Tb*TIF2-F2H are 100%, 82%, and 72% at 0, 24, and 40 hrs, respectively), *Tb*TRF protein levels were not decreased (Fig 1C), indicating that *Tb*TRF is degraded by the proteasome when *Tb*TIF2 is absent. As a control, we also examined the protein levels of *Tb*PSA6 and *Tb*AUK1. *Tb*PSA6 is the A6 subunit of the 20S proteasome and is not degraded by the proteasome [46], while *Tb*AUK1, the *T*. *brucei* Aurora like kinase, is degraded by the proteasome [47]. As expected, adding MG-132 blocks the activity of the proteasome and the *Tb*AUK1 protein level was higher in these cells than in cells not treated with MG-132 (Fig 1C). In contrast, the *Tb*PSA6 protein level was not affected by MG-132 (Fig 1C). In this particular induction, the *Tb*AUK1 protein level appears to be slightly increased upon *Tb*TIF2 depletion in the absence of MG-132 (Fig 1C). To better examine the effect of *Tb*TIF2 depletion on *Tb*AUK1 protein levels, we repeated the induction three more times and quantified the *Tb*AUK1-3HA protein levels in Adobe Photoshop using tubulin or EF-2 as a loading control. On average (calculated from four independent inductions), *Tb*AUK1-3HA protein levels changed from 100% at 0 hr to 106.8% at 24 hrs and 103.8% at 40 hrs after induction, indicating that *Tb*TIF2 does not affect *Tb*AUK1 protein levels significantly. Therefore, *Tb*TIF2 regulates *Tb*TRF protein levels by inhibiting its degradation by the proteasome.

We also examined both *Tb*TRF and *Tb*TIF2 protein levels in *Tb*TRF RNAi cells. Upon RNAi induction, we only observed depletion of *Tb*TRF, while the *Tb*TIF2 protein level was stable for at least 24 hrs. The mild decrease in *Tb*TIF2 protein levels after 36 hrs of *Tb*TRF RNAi induction is likely due to the severe growth defect in *Tb*TRF-depleted cells [29].

### *Tb*TRF does not affect the amount of subtelomere DNA double strand breaks (DSBs)

**Fig 2:**
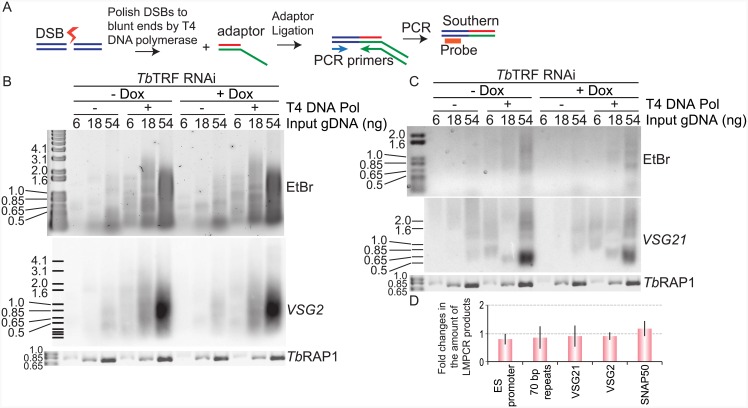
*Tb*TRF depletion does not affect the amount of DSBs inside VSG ESs. (A) Principle of LMPCR assay. After DSBs (represented by a bolt) form, an adapter is ligated with the genomic DNA at the break sites if they have blunt ends. Treating genomic DNA with T4 DNA polymerase converts staggered broken ends into blunt ends. The ligated products are then amplified by PCR using a locus-specific forward primer and the adapter-specific reverse primer. The PCR amplified products are subsequently detected by locus-specific probes in Southern analysis. (B & C) LMPCR analyses were performed in *Tb*TRF RNAi cells. The LMPCR products were hybridized with *VSG2* (B) and *VSG21* (C). In panels B & C, the Ethidium Bromide (EtBr)-stained LMPCR products are shown at the top, the Southern blot result is shown in the middle, and the PCR products using primers specific to the *TbRAP1* gene (as a loading control) are shown at the bottom. The amounts of input genomic DNA, either treated (+) or not treated (−) with T4 DNA polymerase, were marked on top of each lane. (D) Quantification of the change in the amounts of LMPCR products (with T4 DNA pol treatment using 54 ng input gDNA) from three independent experiments. Average values are shown. Error bars represent standard deviations.

The fact that depletion of *Tb*TIF2 leads to depletion of *Tb*TRF further suggests that *Tb*TIF2 and *Tb*TRF function in the same pathway to influence VSG switching and allow normal cell proliferation. To further investigate this possibility, we tested whether depletion of *Tb*TRF results in more subtelomeric DSBs as we observed in *Tb*TIF2 RNAi cells. Due to extremely limited availability of specific antibodies against γH2A, which has been shown to be deposited at the chromatin with DSBs [48], we used Ligation-mediated PCR (LMPCR) analysis (Fig 2A) to detect and estimate the amount of DSBs in active and silent VSG ESs as we did previously [19]. LMPCR has a higher resolution than γH2A Immunofluoresence analysis (IF) so that we can determine whether DSBs are in subtelomeric VSG ESs, while IF can only reveal whether DSBs are in telomere vicinity in general (can be either telomeric or subtelomeric or both). Surprisingly, although DSBs were still detected, the amount of DSBs within *VSG* ESs was not increased when *Tb*TRF was depleted (Fig 2). We examined the single-copy active *VSG2* (Fig 2B) and silent *VSG21* (Fig 2C) and multi-copy ES promoter (S1A Fig) and 70 bp repeat regions (S1B Fig), and the results are reproducible. Quantification of LMPCR product levels indicated that there is no significant change observed in the DSB levels inside VSG ESs before and after depletion of *Tb*TRF (Fig 2D). In addition, depletion of *Tb*TRF did not affect the DSB levels at a chromosome internal *SNAP50* gene locus (S1C Fig). Therefore, depletion of *Tb*TIF2 appears to have two independent effects: decreasing *Tb*TRF protein levels and increasing subtelomeric DSB levels.

### Induced expression of an ectopic *Tb*TRF allele slightly improves cell growth in *Tb*TIF2 RNAi cells

**Fig 3:**
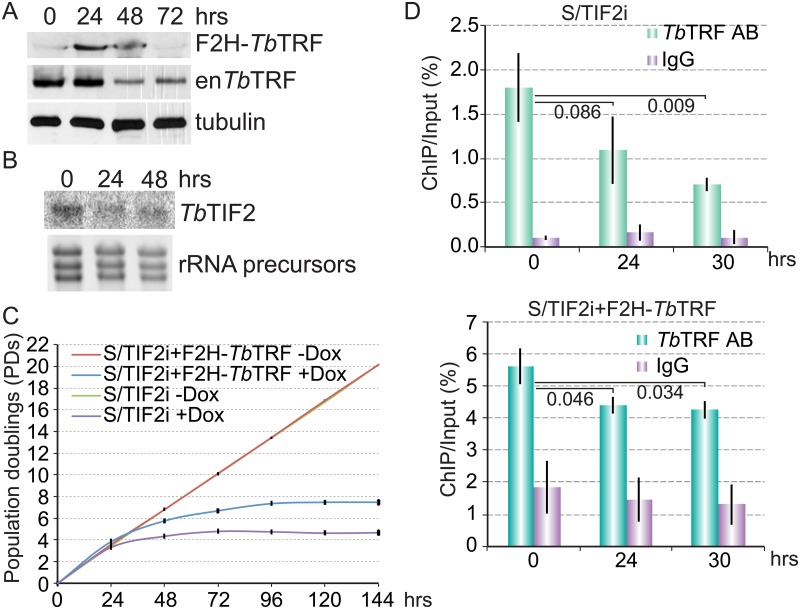
Inducing the expression of an ectopic F2H-*Tb*TRF allele in *Tb*TIF2 RNAi cells. (A) Western analysis showing the expression of the ectopic F2H-*Tb*TRF and eventual depletion of both endogenous and ectopic *Tb*TRF proteins upon depletion of *Tb*TIF2. (B) Northern analysis showing the depletion of *Tb*TIF2 mRNA upon induction of RNAi. rRNA precursors were shown as a loading control. (C) Growth curves show that expression of an ectopic F2H-*Tb*TRF allele slightly improved the cell growth in *Tb*TIF2 RNAi cells. (D) Quantification of ChIP analysis using *Tb*TRF antibody and IgG (as a control) performed in S/TIF2i cells (top) and in S/TIF2i+F2H-*Tb*TRF cells (bottom). ChIP products were hybridized with a TTAGGG repeat probe. As a control, ChIP products were also hybridized with a 50 bp repeat (located upstream of ES promoters) probe, and the quantification results are shown in S2 Fig. Average values were calculated from three independent experiments. Error bars represent standard deviations. Numbers represent P values (unpaired t-test) between groups of values as indicated.

To further investigate the functional relationship between *Tb*TRF and *Tb*TIF2 in maintaining subtelomere stability and in cell survival, we introduced an ectopic F2H-tagged WT allele of *Tb*TRF into the *Tb*TIF2 RNAi strain used previously for examining *Tb*TIF2’s effect on VSG switching, S/TIF2i [19]. We have shown that F2H-*Tb*TRF has essential *Tb*TRF functions and can rescue mutant phenotypes in *Tb*TRF RNAi cells [18,29]. *Tb*TIF2 RNAi and expression of F2H-*Tb*TRF in S/TRFi+F2H-*Tb*TRF cells can be induced simultaneously by adding doxycycline [49]. Upon adding doxycycline, we observed induced F2H-*Tb*TRF expression within the first 48 hours (Fig 3A). However, expression of F2H-*Tb*TRF was not stably maintained 72 hrs after the induction, because *Tb*TIF2 was depleted soon after induction of *Tb*TIF2 RNAi, as shown in northern blotting analysis (Fig 3B). In addition, we also observed that the endogenous *Tb*TRF protein level decreased significantly 48 hrs after inducing *Tb*TIF2 RNAi (Fig 3A). Even so, at 24–48 hrs after induction, the *Tb*TRF protein level in S/TIFi+F2H-*Tb*TRF cells has not significantly decreased as in S/TIF2i cells, suggesting that expression of the ectopic F2H-*Tb*TRF allele resulted in more *Tb*TRF proteins in the cell that presumably require longer time to be completely degraded after removal of *Tb*TIF2.

Consistent with our observation of delayed *Tb*TRF depletion in S/TIF2i+F2H-*Tb*TRF cells, we found that these cells showed mildly better growth than S/TIF2 cells (Fig 3C). In S/TIF2i cells, cell growth slowed down significantly by 24 hrs and was completely arrested by 72 hrs after induction of *Tb*TIF2 RNAi, while in S/TIF2i+F2H-*Tb*TRF cells, cell growth slowed down by 48 hrs and was completely arrested by 96 hrs after induction. Therefore, maintaining *Tb*TRF protein levels for a longer time slightly delayed the cell growth arrest phenotype in *Tb*TIF2 RNAi cells. This further supports the idea that depletion of *Tb*TIF2 caused two parallel effects that both lead to cell growth arrest.

We previously found that when the DNA binding activity of *Tb*TRF is significantly impaired [less than 20% of *Tb*TRF is associated with the telomeric chromatin in Chromatin IP (ChIP) experiment], the VSG switching frequency is increased [18]. To examine whether *Tb*TRF’s telomere binding is affected in S/TIF2i and S/TIF2i+F2H-*Tb*TRF cells, we performed ChIP analysis using a *Tb*TRF antibody [29]. In S/TIF2i cells, the association between *Tb*TRF and the telomere chromatin decreased significantly upon *Tb*TIF2 depletion (Fig 3D, top), as *Tb*TRF is degraded. In S/TIF2i+F2H-*Tb*TRF cells, within 30 hrs after induction of *Tb*TIF2 RNAi, the association of *Tb*TRF with the telomeric DNA was decreased mildly, but ~75% of *Tb*TRF still remained at the telomere (Fig 3D, bottom), indicating that expression of the ectopic F2H-*Tb*TRF helped to keep sufficient *Tb*TRF at the telomere within a short frame of time after *Tb*TIF2 was depleted. This mild decrease of *Tb*TRF binding at the telomere is not expected to affect VSG switching frequencies based on our previous observations [18].

### Expression of ectopic *Tb*TRF does not suppress the phenotype of more subtelomeric DSBs in *Tb*TIF2 RNAi cells

**Fig 4:**
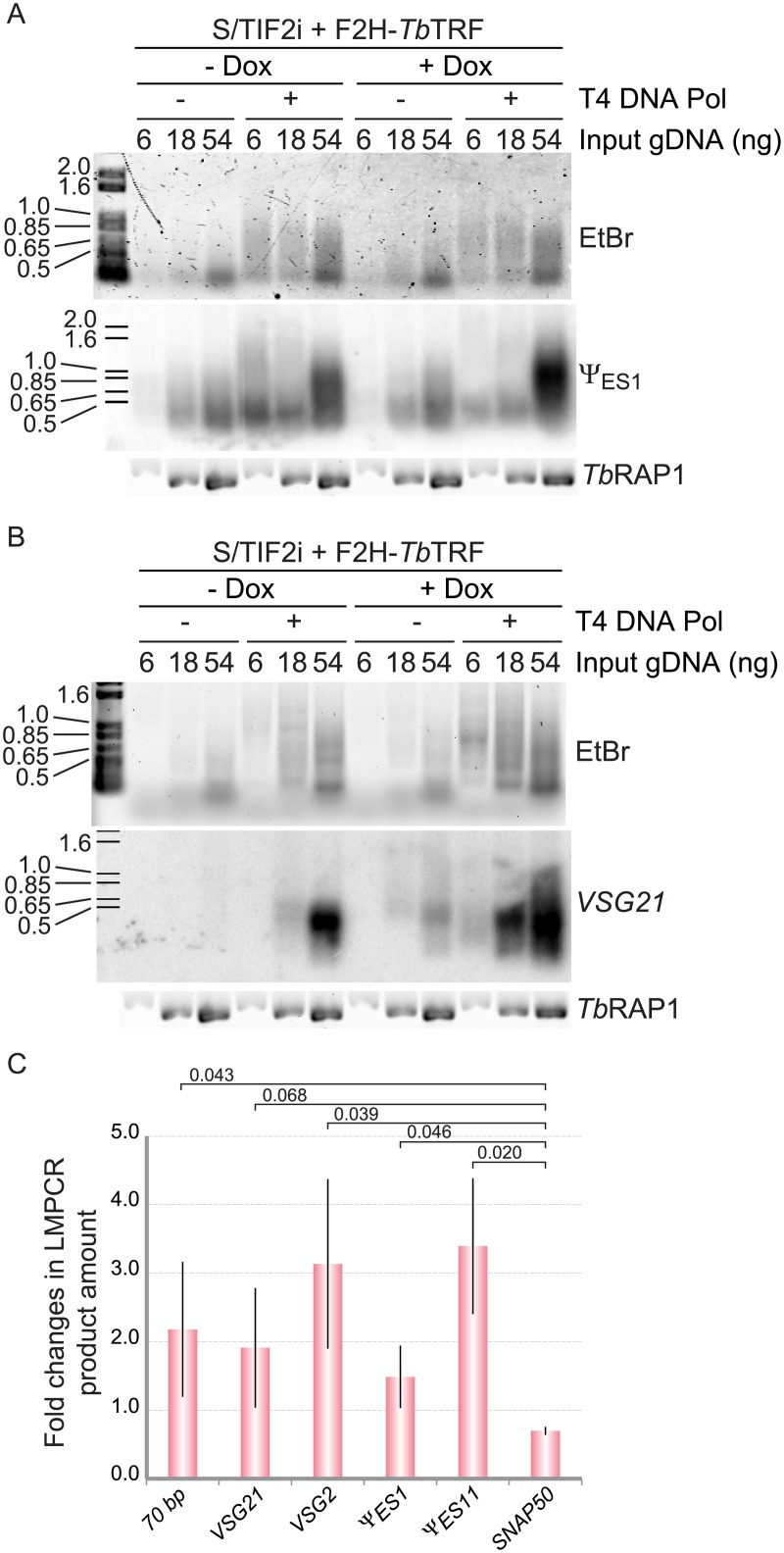
Expression of ectopic F2H-*Tb*TRF does not suppress the phenotype of increased subtelomeric DSB numbers in *Tb*TIF2 RNAi cells. LMPCR analyses were performed in S/TIF2i+F2H-*Tb*TRF cells. LMPCR products were hybridized with *VSG pseudogene* ***ψ***_*ES1*_ (A) and *VSG21* (B). Ethidium Bromide (EtBr)-stained LMPCR products are shown at the top, the Southern blot result is shown in the middle, and the PCR products using primers specific to the *TbRAP1* gene (as a loading control) are shown at the bottom. (C) Quantification of the change in the amounts of LMPCR products (with T4 DNA pol treatment using 54 ng input gDNA) from three independent experiments. Average values are shown. Error bars represent standard deviations. Numbers represent P values of unpaired t-tests between pairs of data groups as indicated.

We found that depletion of *Tb*TRF does not increase the subtelomeric amount of DSBs (Fig 2), suggesting that more subtelomeric DSBs in *Tb*TIF2-depleted cells are independent of decreased *Tb*TRF protein levels. If this is the case, expression of the ectopic F2H-*Tb*TRF in *Tb*TIF2 RNAi cells will not suppress the phenotype of increased DSB levels. To confirm this, we performed the LMPCR analysis (Fig 2A) in S/TIF2i+F2H-*Tb*TRF cells to detect DSBs at subtelomeres. Indeed, 24 hrs after induction of *Tb*TIF2, when *Tb*TRF level had not decreased significantly and more than 75% of *Tb*TRF were still associated with the telomere, we observed a significant increase in the amount of subtelomeric DSBs (Fig 4) the same as in S/TIF2i cells [19]. We tested the active *VSG2* (S3A Fig) and ***ψ***_*ES1*_ pseudogene (Fig 4A), the silent *VSG21* (Fig 4B) and ***ψ***_*ES11*_ pseudogene (data not shown), and 70 bp repeats (S3B Fig) and observed similar results at all tested loci. To compare the fold change in DSB levels in S/TIF2i+F2H-*Tb*TRF cells with that in S/TIF2i cells [19], we also quantified the fold change in DSB levels at multiple gene loci including the active *VSG2* and ***ψ***_*ES1*_ loci, the silent *VSG21* and ***ψ***_*ES11*_ loci, 70 bp repeats, and the chromosome internal *SNAP50* locus (Fig 4C). In S/TIF2i+F2H-*Tb*TRF cells, depletion of *Tb*TIF2 led to ~ 1.5–3.5 fold increase in subtelomeric DSB levels, which is the same as in S/TIF2i cells [19]. This observation also confirmed that induction of RNAi in S/TIF2i+F2H-*Tb*TRF cells efficiently depleted *Tb*TIF2 as we did previously in S/TIF2i cells.

### Expression of ectopic *Tb*TRF in *Tb*TIF2 RNAi cells partially suppresses the phenotype of increased VSG switching frequency

Our observations suggest that the two effects of *Tb*TIF2 depletion, increased subtelomeric DSB levels and decreased *Tb*TRF protein levels, are independent of each other. Still, both are expected to contribute to subsequent increased VSG switching frequency. To test this, we estimated the VSG switching frequencies in S/TIF2+F2H-*Tb*TRF cells the same way we did previously [18]. Both S/TIF2i and S/TIF2+F2H-*Tb*TRF strains were derived from the HSTB261 strain, in which the active *VSG2*-containing ES are marked with a *Blasticidin resistance* (*BSD*) marker immediately downstream of the ES promoter and a *Puromycin resistance* (*PUR*)–*Thymidine Kinase* (*TK*) fusion gene between the active *VSG2* and the 70 bp repeats [15].

To be able to compare current results with previous observations, we performed the switching assay in S/TIF2i, S/TIF2+F2H-*Tb*TRF, and S/ev control cells that carry an empty RNAi construct, using exactly the same switching protocol as before [18]. Cells were induced for 30 hrs. Then doxycycline was removed by extensive washing [19]. This allows cells to recover from the growth arrest so that VSG switchers can subsequently be obtained. To enrich for VSG switchers, cells were first mixed with MACS beads conjugated with a VSG2 monoclonal antibody. Non-switchers that still express VSG2 are trapped with MACS beads, while switchers were in the flow through fraction, which is further selected by ganciclovir [15]. VSG switchers should no longer express the *TK* gene targeted immediately upstream of the active *VSG2*, and cells not expressing TK are resistant to ganciclovir, a nucleoside analog [50].

**Fig 5:**
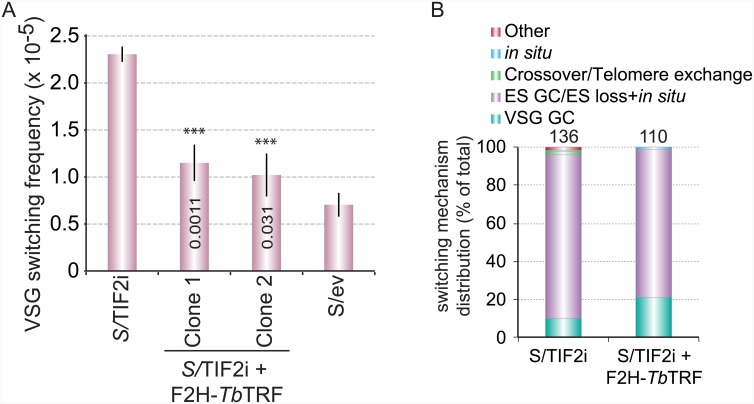
Expressing an ectopic F2H-*Tb*TRF allele partially suppresses the phenotype of increased VSG switching frequency in *Tb*TIF2 RNAi cells. (A) VSG switching frequencies in several strains. Averages were calculated from three independent experiments. Error bars represent standard deviations. Numbers labeled on middle columns are P values (unpaired t-test) calculated between switching frequencies in S/TIF2+F2H-*Tb*TRF cells and that in S/ev cells. Asterisks represent P values (unpaired t-test) calculated between values in S/TIF2+F2H-*Tb*TRF cells and that in S/TIF2i cells. ***, P<0.001. (B) VSG switching mechanisms in different strains. The total number of analyzed switchers in each strain was indicated on top of the corresponding column.

We found that VSG switching frequencies in S/TIF2i+F2H-*Tb*TRF cells are lower than that in S/TIF2i cells but are higher than that in S/ev cells (Fig 5A), supporting the idea that expression of an ectopic F2H-*Tb*TRF allele partially suppressed the phenotype of increased VSG switching frequency in *Tb*TIF2 depleted cells.

By determining the expression status and genotypes of the markers in the obtained VSG switchers, we can reveal the underlying mechanisms of switching events [15,18,19]. Previously, we found that although VSG switchers that lost the original active ES are already the most frequent switching events (70%), depletion of *Tb*TIF2 further increased the percentage of such VSG switchers (80–90%) [19]. Here we estimated the VSG switching frequency in S/TIF2i cells using the MACS-based VSG switching assay and observed the same phenotype, where VSG switchers that lost the original active ES consist of 90% of all switchers (Fig 5B). In addition, expression of the ectopic F2H-*Tb*TRF reduced the percentage of this type of switchers to 80% (Fig 5B), which is between the levels in S/TIF2i cells and S/ev cells [19]. Therefore, examination of VSG switching in S/TIF2i+F2H-*Tb*TRF cells further indicates that expressing F2H-*Tb*TRF and maintaining cellular *Tb*TRF protein levels at the time of performing switching assays partially suppresses phenotypes in *Tb*TIF2 RNAi cells.

## Discussion

Human African trypanosomiasis is fatal without treatment. However, only few drugs are available for treating *T*. *brucei* infections, all with severe side effects. We previously showed that both *Tb*TIF2 and *Tb*TRF are essential for normal cell proliferation and have weak sequence homology with their mammalian homologues only within functional domains [19,29]. Therefore, *Tb*TIF2 and *Tb*TRF could serve as anti-parasite drug targets. However, the underlying mechanisms of *Tb*TIF2 and *Tb*TRF functions are unclear, and whether *Tb*TIF2 and *Tb*TRF function in the same genetic pathway is unknown. Our current investigation reveals that the functions of these two telomere proteins are not identical but are partially overlapping.

The mechanisms of *Tb*TIF2 and *Tb*TRF functions are at least partially overlapping. *Tb*TRF interacts with *Tb*TIF2, and both suppress subtelomeric gene conversion events involving the whole active VSG ES [18,19]. More importantly, depletion of *Tb*TIF2 also leads to depletion of *Tb*TRF (Fig 1). Clearly, decreased *Tb*TRF protein levels in *Tb*TIF2-depleted cells is an important contributing factor for the growth defect and increased VSG switching frequency, as *Tb*TRF is essential for normal cell growth and also suppresses VSG switching [18,29].

*Tb*TIF2 has an additional important function in maintaining subtelomere integrity [19], which we now show is independent of *Tb*TRF. Depletion of *Tb*TRF does not affect the amount of DSBs inside VSG ESs (Fig 2); expressing an ectopic WT allele of *Tb*TRF in S/TIF2i cells does not suppress the phenotype of more subtelomere DSBs but mildly improved the cell growth and partially suppressed the phenotype of increased VSG switching frequency. Therefore, functions of *Tb*TIF2 and *Tb*TRF are not identical.

Based on our observations, we conclude that the functional interaction pathways between *Tb*TIF2 and *Tb*TRF are as follows: *Tb*TIF2 depletion has two independent effects: increased amount of subtelomere DSBs and decreased *Tb*TRF protein levels. Both effects are known to contribute to growth defect and increased VSG switching frequencies [18,19,29]. First, DSBs induced in the active VSG ES are detrimental to *T*. *brucei* cells and cause >85% of cell death [28]. At the same time, DSBs induced near the 70 bp repeats upstream of the active *VSG* gene are also a potent inducer for VSG switching [27,28]. Therefore, the increased amount of subtelomere DSBs in *Tb*TIF2 RNAi cells is an important contributor for cell growth arrest and increased VSG switching frequency. Second, *Tb*TRF is known to have an essential role in maintaining the terminal telomere structure, which is likely the reason why it is essential for normal cell proliferation [29]. *Tb*TRF also suppresses subtelomere gene conversion events that lead to VSG switching [18]. Therefore, decreased *Tb*TRF protein levels induced by *Tb*TIF2 depletion are also an important factor contributing to an acute elevated VSG switching frequency and eventual growth arrest.

It is interesting that *Tb*TIF2 is essential for maintaining subtelomere integrity, while its interacting factor *Tb*TRF is not. Since *Tb*TRF binds the duplex telomere DNA directly [29], it is possible that *Tb*TRF is more important for maintaining telomere than subtelomere integrity. Our previous observation that *Tb*TRF is important for maintaining the terminal telomere structure is consistent with this view [29]. Mammalian TRF2, the functional homologue of *Tb*TRF [29], is also essential for maintaining the telomere terminal G-overhang structure and for preventing chromosome end-to-end fusions through the non-homologous end-joining (NHEJ) pathway [51–53]. We speculate that loss of *Tb*TRF may also result in chromosome end fusions and subsequent breakage-fusion-bridge cycle, which often result in large terminal chromosome deletions [54]. Because VSG is essential, if the chromosome end containing the active *VSG* ES is lost, then only VSG switchers can survive. As a consequence, *Tb*TRF depletion would result in increased VSG switching [18]. In this case, the DNA break sites can locate well upstream of the active *VSG* ES, hence were not detected in the LMPCR analysis where ES-specific probes were used. Although the essential player in NHEJ, DNA ligase IV, has not been identified in *T*. *brucei* [55], micro-homology mediated end-joining has been observed in *T*. *brucei* [55,56], which can also mediate telomere end fusions. Due to the fact that *T*. *brucei* chromosomes do not condense during mitosis and the limited sensitivity of our current molecular tools, telomere end-to-end fusions have not been detected in *T*. *brucei* yet. Developing more sensitive molecular tools would help their detection in the future.

Previous studies indicated that DSBs downstream of the active *VSG* gene mostly lead to switchers that lost the whole active ES [28]. Therefore, it is also possible that *Tb*TRF depletion may cause DSBs only in the telomere repeats that lead to the loss of the active *VSG* ES and subsequent VSG switching. Although these cells have telomerase activity that can elongate telomere sequences after telomere breaks [57,58], it has been shown that the active telomere are more prone to large telomere truncations than silent telomeres [59], presumably because the active telomere is expressed [60], which make it more fragile [61]. DSBs inside the telomere repeats may not be easily detected by the LMPCR analysis, as the most telomere-proximal locus-specific probe is located at the 5’ end of the *VSG* gene, and DSBs located a few kilobases downstream may not be efficiently detected by LMPCR due to limited PCR efficiency.

Although we have identified *Tb*RAP1 as another *Tb*TRF interacting telomere protein [42], *Tb*RAP1, but not *Tb*TRF or *Tb*TIF2, is essential for subtelomeric VSG silencing [19,42,62]. In addition, we have not detected any direct interaction between *Tb*RAP1 and *Tb*TIF2. Furthermore, in yeast 2-hybrid analysis, *Tb*RAP1 interacts with *Tb*TRF weakly [42], while *Tb*TIF2 interacts with *Tb*TRF very strongly [19]. Therefore, we anticipate that *Tb*TIF2 and *Tb*TRF function independently from *Tb*RAP1.

Depletion of *Tb*TRF does not increase the amount of DSBs in subtelomeric ESs, while depletion of *Tb*TIF2 does, indicating that *Tb*TIF2 has essential functions independent of *Tb*TRF. Therefore, it would be interesting to investigate whether recruiting *Tb*TIF2 to telomeres is completely dependent on *Tb*TRF, as *Tb*TRF has a duplex telomere DNA binding activity [29] while *Tb*TIF2 does not have any functional domain suggesting DNA binding activity [19]. Although we have not identified any telomere proteins other than *Tb*TRF [29], *Tb*TIF2 [19], and *Tb*RAP1 [42] in *T*. *brucei*, it is very possible that the *T*. *brucei* telomere complex has more than just three protein members. For example, *T*. *brucei* has a telomere G-rich 3’ overhang at the very end [63]. Although this overhang is short, we expect it to be bound and protected by proteins with single stranded DNA binding activity. It has been shown that Leishmania RPA1 can bind telomeric single stranded DNA [64]. Since *T*. *brucei* and Leishmania are closely related, it is possible that *T*. *brucei* RPA1 can also bind telomeric G-overhang. If *Tb*TIF2 interacts with single-stranded telomere DNA binding factors, it can also be recruited to the telomere independently of *Tb*TRF.

Our studies further validate that both *Tb*TIF2 and *Tb*TRF are essential for normal *T*. *brucei* proliferation. Since these proteins are only weakly homologous to their mammalian counterparts [19,29], they are good potential targets for anti-parasite agents. In addition, our results indicate that targeting *Tb*TIF2 would be more efficient than targeting *Tb*TRF, as *Tb*TIF2 depletion causes simultaneous depletion of *Tb*TRF in addition to increased subtelomeric DSB numbers. In human cells, TIN2 stabilizes TRF1 proteins by preventing Tankyrase 1 from modifying TRF1 [35,36], which in turn prevents TRF1 from being ubiquitinated by SCF^FBX4^ [38] and subsequent degradation by the 26S proteasome [38,39]. However, tankyrase 1 and SCF^FBX4^ homologs have not been identified in *T*. *brucei*, and whether *Tb*TRF is ubiquitinated before it is degraded by the proteasome is unknown. Further studies on *Tb*TRF modifications will help to reveal the detailed mechanisms of how *Tb*TIF2 regulates *Tb*TRF protein levels.

## Supporting Information

S1 Fig LMPCR analysis of subtelomeric DSBs in *Tb*TRF RNAi cells. (EPS)

S2 Fig Control ChIP experiments to examine association of *Tb*TRF and the 50 bp repeats. (EPS)

S3 Fig LMPCR analysis of subtelomeric DSBs in S/TIFi+F2H-*Tb*TRF cells. (EPS)

S1 Table S/TIF2i Switcher phenotype and genotype characterization. (DOCX)

S2 Table S/TIF2i+F2H-*Tb*TRF Switcher phenotype and genotype characterization. (DOCX)

S3 Table List of primers used in this study. (DOCX)

S1 Procedure Supplemental Experimental Procedure. (PDF)
