# Supplementary material for: Trypanosoma brucei TIF2 and TRF Suppress VSG Switching Using Overlapping and Independent Mechanisms
Source: PLoS One. 2016 Jun 3;11(6):e0156746. doi: 10.1371/journal.pone.0156746 (PMC4892550; doi:10.1371/journal.pone.0156746)
Supplement: S1 Table — (DOCX) [file pone.0156746.s004.docx]

**S1 Table. S/TIF2i Switcher phenotype and genotype characterization.**

| **Switchers ID** | **Puromycin 2 µg/ml** | **Blasticidin 5 µg/ml** | **Blasticidn 100 µg/ml** | **VSG2 PCR** | **BSD PCR** | **Switching mechanism** |
| --- | --- | --- | --- | --- | --- | --- |
| 1 | S | S | S | - | - | ES GC/ES Loss+In Situ |
| 2 | S | S | S | - | - | ES GC/ES Loss+In Situ |
| 3 | S | S | S | - | - | ES GC/ES Loss+In Situ |
| 4 | S | S | S | - | - | ES GC/ES Loss+In Situ |
| 5 | S | S | S | - | - | ES GC/ES Loss+In Situ |
| 6 | S | R | R | - | + | VSG GC |
| 7 | S | S | S | - | - | ES GC/ES Loss+In Situ |
| 8 | S | S | S | - | - | ES GC/ES Loss+In Situ |
| 9 | S | S | S | - | - | ES GC/ES Loss+In Situ |
| 10 | S | S | S | - | - | ES GC/ES Loss+In Situ |
| 11 | S | S | S | - | - | ES GC/ES Loss+In Situ |
| 12 | S | R | R | - | + | VSG GC |
| 13 | S | S | S | - | - | ES GC/ES Loss+In Situ |
| 14 | S | S | S | - | - | ES GC/ES Loss+In Situ |
| 15 | S | S | S | - | - | ES GC/ES Loss+In Situ |
| 16 | S | S | S | - | - | ES GC/ES Loss+In Situ |
| 17 | S | S | S | - | - | ES GC/ES Loss+In Situ |
| 18 | S | S | S | - | - | ES GC/ES Loss+In Situ |
| 19 | S | S | S | - | - | ES GC/ES Loss+In Situ |
| 20 | S | R | R | - | + | VSG GC |
| 21 | S | S | S | - | - | ES GC/ES Loss+In Situ |
| 22 | S | S | S | - | - | ES GC/ES Loss+In Situ |
| 23 | S | S | S | - | - | ES GC/ES Loss+In Situ |
| 24 | S | S | S | + | - | OTHER |
| 25 | S | S | S | - | - | ES GC/ES Loss+In Situ |
| 26 | S | S | S | - | - | ES GC/ES Loss+In Situ |
| 27 | S | S | S | - | - | ES GC/ES Loss+In Situ |
| 28 | S | S | S | - | - | ES GC/ES Loss+In Situ |
| 29 | S | S | S | - | - | ES GC/ES Loss+In Situ |
| 30 | S | S | S | - | - | ES GC/ES Loss+In Situ |
| 31 | S | S | S | - | - | ES GC/ES Loss+In Situ |
| 32 | S | S | S | - | - | ES GC/ES Loss+In Situ |
| 33 | S | R | R | - | + | VSG GC |
| 34 | S | S | S | - | - | ES GC/ES Loss+In Situ |
| 35 | S | S | S | - | - | ES GC/ES Loss+In Situ |
| 36 | S | R | R | - | + | VSG GC |
| 37 | S | R | R | + | + | Crossover |
| 38 | S | S | S | - | - | ES GC/ES Loss+In Situ |
| 39 | S | R | R | - | + | VSG GC |
| 40 | S | S | S | - | - | ES GC/ES Loss+In Situ |
| 41 | S | S | S | - | - | ES GC/ES Loss+In Situ |
| 42 | S | R | R | - | + | VSG GC |
| 43 | S | S | S | - | - | ES GC/ES Loss+In Situ |
| 44 | S | S | S | - | - | ES GC/ES Loss+In Situ |
| 45 | S | S | S | - | - | ES GC/ES Loss+In Situ |
| 46 | S | R | R | - | + | VSG GC |
| 47 | S | S | S | - | - | ES GC/ES Loss+In Situ |
| 48 | S | R | R | - | + | VSG GC |
| 49 | S | S | S | - | - | ES GC/ES Loss+In Situ |
| 50 | S | R | R | + | + | Crossover |
| 51 | S | S | S | - | - | ES GC/ES Loss+In Situ |
| 52 | S | R | R | - | + | VSG GC |
| 53 | S | R | R | - | + | VSG GC |
| 54 | S | S | S | - | - | ES GC/ES Loss+In Situ |
| 55 | S | S | S | - | - | ES GC/ES Loss+In Situ |
| 56 | S | S | S | - | - | ES GC/ES Loss+In Situ |
| 57 | S | S | S | - | - | ES GC/ES Loss+In Situ |
| 58 | S | S | S | - | - | ES GC/ES Loss+In Situ |
| 59 | S | S | S | - | - | ES GC/ES Loss+In Situ |
| 60 | S | S | S | - | - | ES GC/ES Loss+In Situ |
| 61 | S | S | S | - | - | ES GC/ES Loss+In Situ |
| 62 | S | S | S | - | - | ES GC/ES Loss+In Situ |
| 63 | S | S | S | - | - | ES GC/ES Loss+In Situ |
| 64 | S | S | S | - | - | ES GC/ES Loss+In Situ |
| 65 | S | S | S | - | - | ES GC/ES Loss+In Situ |
| 66 | S | S | S | - | - | ES GC/ES Loss+In Situ |
| 67 | S | S | S | - | - | ES GC/ES Loss+In Situ |
| 68 | S | S | S | - | - | ES GC/ES Loss+In Situ |
| 69 | S | S | S | - | - | ES GC/ES Loss+In Situ |
| 70 | S | S | S | - | - | ES GC/ES Loss+In Situ |
| 71 | S | S | S | - | - | ES GC/ES Loss+In Situ |
| 72 | S | S | S | - | - | ES GC/ES Loss+In Situ |
| 73 | S | R | R | - | + | VSG GC |
| 74 | S | S | S | - | - | ES GC/ES Loss+In Situ |
| 75 | S | S | S | - | - | ES GC/ES Loss+In Situ |
| 76 | S | S | S | - | - | ES GC/ES Loss+In Situ |
| 77 | S | S | S | - | - | ES GC/ES Loss+In Situ |
| 78 | S | S | S | - | - | ES GC/ES Loss+In Situ |
| 79 | S | S | S | - | - | ES GC/ES Loss+In Situ |
| 80 | S | S | S | - | - | ES GC/ES Loss+In Situ |
| 81 | S | S | S | - | - | ES GC/ES Loss+In Situ |
| 82 | S | S | S | - | - | ES GC/ES Loss+In Situ |
| 83 | S | R | S | + | + | *in situ* |
| 84 | S | S | S | - | - | ES GC/ES Loss+In Situ |
| 85 | S | S | S | - | - | ES GC/ES Loss+In Situ |
| 86 | S | S | S | - | - | ES GC/ES Loss+In Situ |
| 87 | S | S | S | - | - | ES GC/ES Loss+In Situ |
| 88 | S | S | S | - | - | ES GC/ES Loss+In Situ |
| 89 | S | S | S | - | - | ES GC/ES Loss+In Situ |
| 90 | S | S | S | - | - | ES GC/ES Loss+In Situ |
| 91 | S | S | S | - | - | ES GC/ES Loss+In Situ |
| 92 | S | S | S | - | - | ES GC/ES Loss+In Situ |
| 93 | S | S | S | - | - | ES GC/ES Loss+In Situ |
| 94 | S | S | S | - | - | ES GC/ES Loss+In Situ |
| 95 | S | S | S | - | - | ES GC/ES Loss+In Situ |
| 96 | S | S | S | - | - | ES GC/ES Loss+In Situ |
| 97 | S | S | S | - | - | ES GC/ES Loss+In Situ |
| 98 | S | S | S | - | - | ES GC/ES Loss+In Situ |
| 99 | S | S | S | - | - | ES GC/ES Loss+In Situ |
| 100 | S | S | S | - | - | ES GC/ES Loss+In Situ |
| 101 | S | S | S | - | - | ES GC/ES Loss+In Situ |
| 102 | S | S | S | - | - | ES GC/ES Loss+In Situ |
| 103 | S | S | S | - | - | ES GC/ES Loss+In Situ |
| 104 | S | S | S | - | - | ES GC/ES Loss+In Situ |
| 105 | S | S | S | - | - | ES GC/ES Loss+In Situ |
| 106 | S | S | S | - | - | ES GC/ES Loss+In Situ |
| 107 | S | S | S | - | - | ES GC/ES Loss+In Situ |
| 108 | S | S | S | - | - | ES GC/ES Loss+In Situ |
| 109 | S | S | S | - | - | ES GC/ES Loss+In Situ |
| 110 | S | S | S | - | - | ES GC/ES Loss+In Situ |
| 111 | S | S | S | - | - | ES GC/ES Loss+In Situ |
| 112 | S | S | S | - | - | ES GC/ES Loss+In Situ |
| 113 | S | S | S | - | - | ES GC/ES Loss+In Situ |
| 114 | S | S | S | + | - | OTHER |
| 115 | S | S | S | - | - | ES GC/ES Loss+In Situ |
| 116 | S | S | S | - | - | ES GC/ES Loss+In Situ |
| 117 | S | S | S | - | - | ES GC/ES Loss+In Situ |
| 118 | S | S | S | - | - | ES GC/ES Loss+In Situ |
| 119 | S | S | S | - | - | ES GC/ES Loss+In Situ |
| 120 | S | S | S | - | - | ES GC/ES Loss+In Situ |
| 121 | S | S | S | - | - | ES GC/ES Loss+In Situ |
| 122 | S | S | S | - | - | ES GC/ES Loss+In Situ |
| 123 | S | S | S | - | - | ES GC/ES Loss+In Situ |
| 124 | S | S | S | - | - | ES GC/ES Loss+In Situ |
| 125 | S | S | S | - | - | ES GC/ES Loss+In Situ |
| 126 | S | S | S | - | - | ES GC/ES Loss+In Situ |
| 127 | S | S | S | - | - | ES GC/ES Loss+In Situ |
| 128 | S | S | S | - | - | ES GC/ES Loss+In Situ |
| 129 | S | S | S | - | - | ES GC/ES Loss+In Situ |
| 130 | S | R | R | - | + | VSG GC |
| 131 | S | S | S | - | - | ES GC/ES Loss+In Situ |
| 132 | S | S | S | - | - | ES GC/ES Loss+In Situ |
| 133 | S | S | S | - | - | ES GC/ES Loss+In Situ |
| 134 | S | S | S | - | - | ES GC/ES Loss+In Situ |
| 135 | S | S | S | - | - | ES GC/ES Loss+In Situ |
| 136 | S | S | S | - | - | ES GC/ES Loss+In Situ |
