# Supplementary material for: Trypanosoma brucei TIF2 and TRF Suppress VSG Switching Using Overlapping and Independent Mechanisms
Source: PLoS One. 2016 Jun 3;11(6):e0156746. doi: 10.1371/journal.pone.0156746 (PMC4892550; doi:10.1371/journal.pone.0156746)
Supplement: S3 Table — (DOCX) [file pone.0156746.s006.docx]

**S3 Table. List of primers used in this study.**

| Primer Name | Primer Sequence |
| --- | --- |
| Linker short | 5'- GTGAATTCAGATC -3' |
| Linker long | 5'- GCGGTGACCCGGGAGATCTGAATTCAC -3' |
| OBL-BeforeESPr-LMPCR-FW | 5'- AGAAATCTCGGATATCAGACTCAC -3' |
| OBL-VSG pseudogene -FW | 5'- ACCGGAACTGCAGGAACAAATG -3' |
| OBL-LMPCR-TEL122-VSGpseudo-unique-FW | 5'- GAAAAGGCTAAGACGCTAAGAATC -3' |
| OBL-SNAP50-unique-LMPCR-FW | 5’- TGAACATTTCAAAGAATGCATTTTTCTTTATTGGA -3’ |
| BES oligo probe | 5'- GACCGTTGTCGGTTCATGCATGACTTTAATCTCATC -3' |
| BES1 VSG pseudogene oligo probe | 5'- GAATACAAAGATGGATGCAAATGGGAAGGCGAAACT -3' |
| 70 bp oligo probe | 5'- ATACGAATATTATAATAAGAGCAGTA -3' |
| BES11 VSG pseudogen oligo probe | 5'- GACGCAGAAGAAGACACCAATAACACAAAC -3' |
| SNAP50 oligo probe | 5’- GGATGGTGCAACCATTACTTTTACCTTAGTTCAG-3’ |
| OBL-*Tb*TRF-start-XhoI-FW | 5’- GCGC CTC GAG ATG TAC TGT CAC GCT GGC GT -3’ |
| OBL-*Tb*TRF-end-BamHI-BW | 5’- GCGC GAT CCT CAC TCG TTA TTC TCC ATA TTG -3’ |
