# Supplementary material for: Trypanosoma brucei TIF2 and TRF Suppress VSG Switching Using Overlapping and Independent Mechanisms
Source: PLoS One. 2016 Jun 3;11(6):e0156746. doi: 10.1371/journal.pone.0156746 (PMC4892550; doi:10.1371/journal.pone.0156746)
Supplement: S1 Procedure — (PDF) [file pone.0156746.s007.pdf]

Supplemental Experimental Procedures

S1 Procedure. Plasmid.

pLew111-F2H-*Tb*TRF-hygro: The Full-length *TbTRF* gene was PCR amplified from WT *T. brucei* genomic DNA using primers OBL-*Tb*TRF-start-XhoI-FW and OBL-*Tb*TRF-end-BamHI-BW and inserted into pLew111-F2H-*Tb*RAP1-BSD [42] to replace the *TbRAP1* gene. The *BSD* gene was subsequently replaced by the hygromycin resistance gene.

S2 Procedure. *T. brucei* cells and culture.

Bloodstream form *T. brucei* Lister 427 cells expressing the T7 polymerase and the Tet repressor (SM) [49] were used for these studies. SM-derived HSTB261 [15] was used to establish strains used for switching assays. S/TIF2i and S/ev strains were described previously [19]. 2/TIF2i and *Tb*TRF RNAi strains established in the SM background were described previously [19,29]. All BF *T. brucei* cells were cultured in HMI-9 medium supplemented with 10% FBS and appropriate antibiotics.

S/TIF2i cells were transfected with EcoRV digested pLew111-F2H-*Tb*TRF-hygro to establish the S/TIF2i+F2H-*Tb*TRF strain. Two clones C17 and C18 were analyzed for their growth, *Tb*TIF2 protein levels, and endogenous and ectopic *Tb*TRF protein levels after induction. Data presented here were from the C18 clone.

S3 Procedure. VSG switching assay using MACS.

Switching assays were performed according to [18]. Briefly, 54,000 cells were inoculated in 38 ml of medium in the absence of puromycin and blasticidin. When cell concentration reached 1.7 million cells/ml (~10.2 population

doublings), a small fraction of cells were plated directly in the absence of GCV to determine plating efficiency. To enrich switchers, 50 million cells were incubated with 10 μl of monoclonal VSG221 antibody [41] for 15 minutes on ice. Cells were washed with the medium once before mixing with 100 μl MACS beads (Goat anti-mouse IgM Microbeads, Miltenyi Biotec) for 15 minutes on ice. Cells were washed once again with the medium and were loaded on to an LD column (Miltenyi Biotec) equilibrated with the medium. The LD column was washed with 2 ml of media and the flow-through fractions containing the switchers were collected (total 2.0-2.5 ml). Collected cells were serially diluted in media containing 4 μg/ml GCV and distributed into 96-well plates. All GCV-resistant clones were subsequently confirmed for their sensitivity to puromycin to exclude GCV-resistant TK mutants. The VSG switching frequency was determined by dividing the number of GCV resistant, puromycin sensitive clones to the total number of input cells (50 million) and normalized to the plating efficiency.

To determine switching mechanisms, switchers were analyzed for 5 μg/ml and 100 μg/ml blasticidin sensitivity. *In situ* switchers are resistant to 5 μg/ml blasticidin but sensitive to 100 μg/ml blasticidin, *VSG* GC and CO switchers are resistant to 100 μg/ml basticidin, and ES GC or ES loss + *in situ* switchers are sensitive to 5 μg/ml blasticidin. To determine the presence of *VSG2* and *BSD* genes, genomic DNA was prepared from switchers and PCR-analyses were performed using *VSG2* and *BSD* specific primers. For selected switchers, the newly expressed VSG were determined by reverse transcribing total RNA using a random primer, PCR using a spliced leader primer and a primer common to all *VSG* genes, and sequencing. Genotypes of these switchers were then confirmed by PFGE of DNA plugs followed by Southern hybridization using *VSG2*, the newly expressed *VSG*, and the *BSD* probes.
